# Supplementary material for: β-Tubulin carboxy-terminal tails exhibit isotype-specific effects on microtubule dynamics in human gene-edited cells
Source: Life Sci Alliance. 2018 Apr 19;1(2):e201800059. doi: 10.26508/lsa.201800059 (PMC6070155; doi:10.26508/lsa.201800059)
Supplement: Supplementary file 1 [file LSA-2018-00059_TableS1.pdf]

Table S1. Effect of the β-tubulin C-terminal tail on microtubule assembly.

	**Untreated**			**Paclitaxel 6 nM**			**Paclitaxel 20 nM**		
**Parameter (mean ± SEM)**	**Full-length βIII-Tubulin (ZB3) (n=150 cells)**	**Truncated βIII-Tubulin (ZB3Δ) (n=133 cells)**	**βIII-tubulin Body + βI-tubulin C-terminal Tail (ZB3/CB1) (n=106 cells)**	**Full-length βIII-Tubulin (ZB3) (n=125 cells)**	**Truncated βIII-Tubulin (ZB3Δ) (n=89 cells)**	**βIII-Tubulin Body + βI-Tubulin C-terminal Tail (ZB3/CB1) (n=100 cells)**	**Full-length βIII-Tubulin (ZB3) (n=113 cells)**	**Truncated βIII-Tubulin (ZB3Δ) (n=99 cells)**	**βIII-Tubulin Body + βI-Tubulin C-terminal Tail (ZB3/CB1) (n=96 cells)**
**Assembly Rate (µm/min)**	26.162 ± 0.061	26.355 ± 0.059	**24.667 ± 0.061**	19.516 ± 0.060*	**21.969 ± 0.093***	**23.622 ± 0.085***	23.311 ± 0.150*	**24.557 ± 0.168***	**25.727±0.210***
**Growth Length (µm)**	1.594 ± 0.032	1.593 ± 0.023	**1.416 ± 0.019**	1.328 ± 0.018*	**1.306 ± 0.033***	**1.210 ± 0.016***	1.177 ± 0.014*	1.130 ± 0.021*	**0.988±0.020***
**Growth Duration (s)**	1.357 ± 0.036	1.284 ± 0.018	**1.153 ± 0.018**	1.701 ± 0.035*	**1.572 ± 0.041***	**1.419 ± 0.029***	1.687 ± 0.045*	**1.518 ± 0.056***	**1.496±0.051***
**Number of microtubule growth events per cell**	333.7 ± 11.64	320.5 ± 10.24	**292.9 ± 10.01**	328.7 ± 9.15	**298 ± 13.87**	**254.7 ± 10.61***	160.1 ± 12.3*	**151.5 ± 16.05***	**125.5±10.19***

Mean ± SEM

Bold: p<0.05 compared with cells expressing the full-length βIII-tubulin protein (ZB3)

* p<0.05 compared with untreated cells
